# Detection of porcine parvovirus using a taqman-based real-time pcr with primers and probe designed for the NS1 gene

**DOI:** 10.1186/1743-422X-7-353

**Published:** 2010-12-02

**Authors:** Cuiping Song, Chao Zhu, Chaofan Zhang, Shangjin Cui

**Affiliations:** 1 Laboratory of Marine Genetics and Breeding (MGB), Ocean University of China, Qingdao 266003, China; 2 China Animal Health and Epidemiology Center, Qingdao 266032, China; 3 Division of Swine Infectious Disease, State Key Laboratory of Veterinary Biotechnology, Harbin Veterinary Research Institute of CAAS, Harbin, China 150001

## Abstract

A TaqMan-based real-time polymerase chain reaction (PCR) assay was devised for the detection of porcine parvovirus (PPV). Two primers and a TaqMan probe for the non-structural protein NS1 gene were designed. The detection limit was 1 × 10^2 ^DNA copies/μL, and the assay was linear in the range of 1 × 10^2 ^to 1 × 10^9 ^copies/μL. There was no cross-reaction with porcine circovirus 2 (PCV2), porcine reproductive and respiratory syndrome virus (PRRSV), pseudorabies virus (PRV), classical swine fever virus (CSFV), or Japanese encephalitis virus (JEV). The assay was specific and reproducible. In 41 clinical samples, PPV was detected in 32 samples with the real-time PCR assay and in only 11 samples with a conventional PCR assay. The real-time assay using the TaqMan-system can therefore be practically used for studying the epidemiology and management of PPV.

## Introduction

Porcine parvovirus (PPV) is an autonomous parvovirus belonging to the genus parvovirus, subfamily *Parvovirinae*, family *Parvoviridae*. It is one of the major etiological agents of reproductive failure in pigs. Reproductive failure caused by PPV is characterized by embryonic and foetal death, mummification, stillbirth, and delayed return to oestrus [1]. In addition, PPV has been implicated as the causative agent of diarrhea, skin disease, and arthritis in swine [2]. PPV has been reported from many different countries [3-5].

PPV is composed of a linear single-stranded segment of DNA approximately 5 kb long (Molitor, T.W., 1983), and its genome has more than two open reading frames (ORF) [6]. The 3' end of ORF1 encodes nonstructural proteins (NS proteins), and the 5' end of ORF2 encodes structural proteins (VP proteins).

For diagnostic purposes, PPV can be rapidly and sensitively detected with polymerase chain reaction (PCR) assays [7,8]. However, current PCR assays for PPV often require multiple steps and do not provide quantitative data. In contrast, real-time PCR using SYBR Green and TaqMan is rapid, specific, and efficient for the large-scale screening, strain identification, and quantification of PPV [9].

NS1, which is encoded by the NS1 gene, is a main nonstructural protein of PPV and is associated with the early and late transcription of the virus. Given that inactivated virus used in current vaccines have only little NS1 protein which could not produce antibody, the presence or absence of antibody against NS1 protein could be used in an NS1-based diagnostic kit for determining in clinical settings whether pigs have been vaccinated with the inactivated-PPV or infected with wild-type PPV, and the test would give a negative result for for vaccinated/noninfected pigs. NS proteins are also important in virus research because they play an important regulatory role in viral replication even though they do not directly participate in the assembly of virus particles.

In this study, a TaqMan-based real-time PCR assay was developed for the rapid and quantitative detection of PPV with a probe specific for the PPV NS1 gene. The results of the real-time PCR assays were compared with those of previously established, conventional PCR assays.

## Materials and methods

### Primers and probes

PCR primers and a TaqMan probe, which were designed with the program DNAStar and synthesized by Saituo Matrix Biotechnology (Haerbin) Co., Ltd, were used to amplify a 123-bp fragment of the NS1 gene. The sequences of the primers and probe were:

NS1-FP (forward primer): 5'-GAAGACTGGATGATGACAGATCCA-3',

NS1-RP (reverse primer): 5'-TGCTGTTTTTGTTCTTGCTAGAGTAA-3'.

NS1-P (probe): FAM-AATGATGGCTCAAACCGGAGGAGA-BHQ1. The probe was labeled with 6-carboxyfluorescein (FAM) at the 5'-end and with BHQ1 at the 3'-end.

### Preparation of standard plasmid DNA

PCR amplification of the NS1 gene was carried out in a reaction mix of 25 μL: 16.0 μL sterilized water, 2.5 μL of 10× buffer, 3.0 μL of dNTP, 1 μL of each primer (NS1-FP and NS1-RP), 1 μL of BQ strain DNA, and 0.5 μL of Ex Taq™ DNA Polymerase (Ex taq). The thermal conditions were as follows: one cycle at 94 C for 5 min; followed by 30 cycles at 94 C for 30 s, 58 C for 45 s, and 72 C for 30 s; with a final extension at 72 C for 7 min.

The PCR product was inserted into a vector, pMD18-T (TaKaRa Biotechnology (Dalian) Co., Ltd.). After the culture was increased in DH5a host bacteria (TaKaRa Biotechnology Co., Ltd), the recombinant plasmid was purified using a commercial test kit (Watson Biotechnologies, Inc.). The products were kept at -20°C for later use.

### Establishment of real-time PCR

The real-time PCR amplifications of the NS1 gene used 25-μL reaction mixtures containing 2.5 μL of 10× buffer, 3.5 μL of dNTP (TaKaRa Biotechnology Co., Ltd), 3 μL of MgCl_2_, 1 μL of each primer (10 pM/μL of NS1-FP and NS1-RP), 1 μL of the recombinant plasmid, 0.5 μL of the probe (PPV-P), 0.5 μL of Hotstar taq (TaKaRa Biotechnology Co., Ltd), and 12.0 μL sterile water. The reactions were carried out in an Rotor-Gene Thermocycler (Corbett Research. Co. Ltd.). The conditions were as follows: one cycle at 95°C for 30 s followed by 40 cycles at 95°c for 10 s, 58°c for 20 s, and 72°C for 20 s. The data were analyzed with the Rotor-Gene software.

### Sensitivity of the real-time PCR

To determine the detection limit and efficiency of the assay, recombinant plasmid of standard DNA was used as a template and was 10-fold serially diluted with sterile water, giving 6.00 × 10^9 ^to 6.00 × 10^1 ^copies/μL. The sensitivity of the real-time PCR was compared with conventional PCR (Yue et al., 2009).

### Specificity of the real-time PCR

To determine the specificity of the real-time PCR, the standard plasmid-positive template and different strains of PPV, five different viruses (PRRSV, CSFV, PRV, JEV, and PCV-2), and control (sterile water) were processed with the real-time PCR.

### Reproducibility of real-time PCR

To determine the reproducibility of the real-time PCR, the standard plasmid was diluted to 6.00 × 10^8^, 6.00 × 10^6^, 6.00 × 10^4^, and 6.00 × 10^2 ^copies/μL. To obtain variation within an assay (within a block), each dilution was processed four different times, i.e., in four blocks, with the real-time PCR assay. Each block contained four repeated determinations for each dilution, giving 16 total determinations for each dilution. Coefficients of variation (CVs) for Ct values within each block and among blocks (using the mean values from each block) were determined.

### Detection of the clinical samples

Forty-one clinical samples (20% organ suspensions stored at -70°C) that were suspected of being infected with PPV were subjected to the real-time PCR and conventional PCR.

## Results

### Establishment of the standard plasmid-positive template

The amplified NS1 gene fragment was about 123 bp long. The plasmid DNA concentration was 0.189 μg/μL before dilution. So, the DNA concentration was equivalent to 6.00 × 10^10 ^copies/μL before dilution.

### Establishment of a standard curve for the real-time PCR

**Figure 1:**
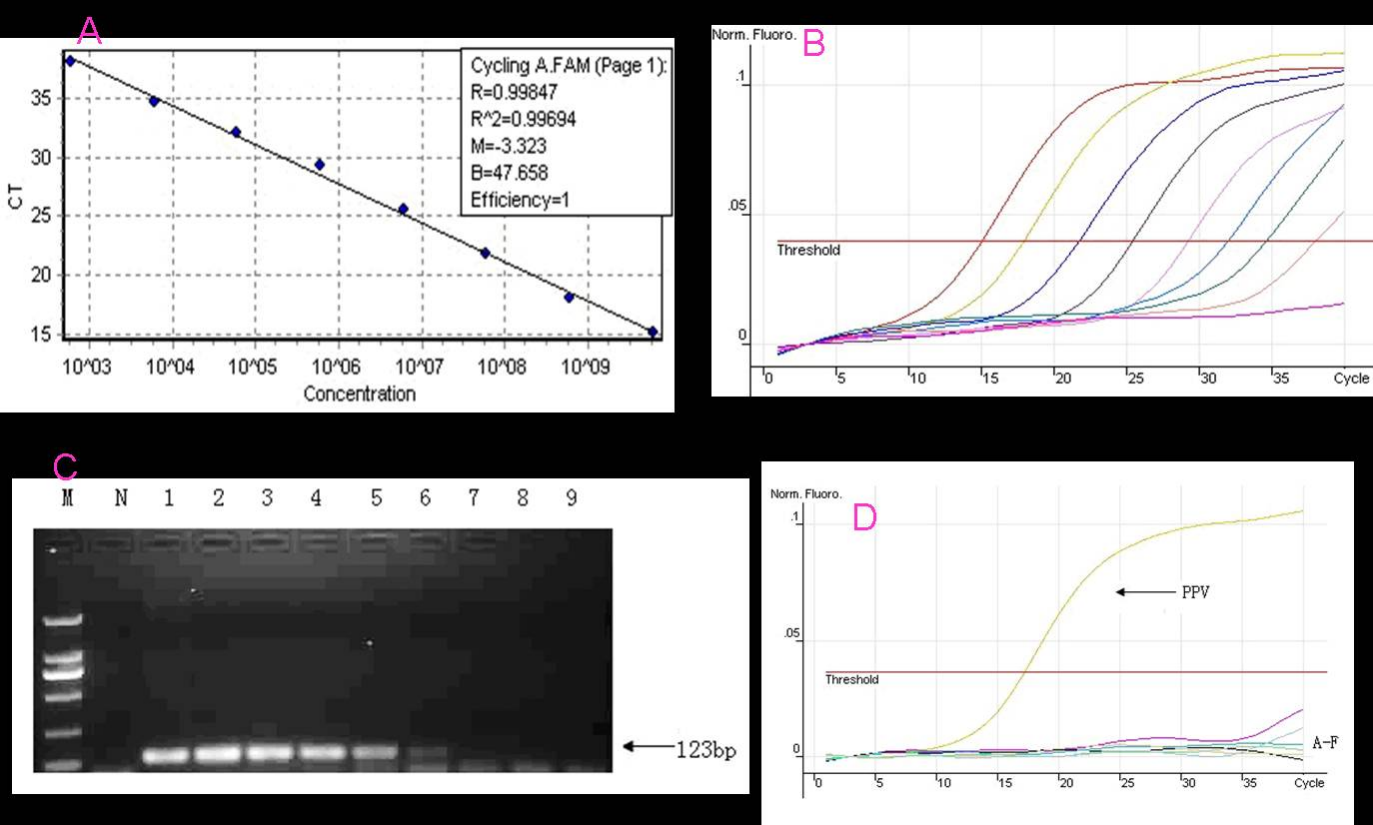
**Establishment of real-time PCR**. A. Standard curves (based on plasmid DNA) indicating the linearity and efficiency for detecting NS1 by real-time PCR. The x-axis represents copies of plasmid DNA in 10-fold dilutions, and the y-axis represents the fluorescence data used for cycle threshold (Ct) determinations. The assays were linear in the range of 10^9 ^to 10^2 ^template copies/μL, with an R^2 ^of 0.996 and a reaction efficiency of 100% for NS1. B. The results of the Quantitation data for Cycling A. FAM. The amplification product was about 123 bp long, and no false amplification was observed. The detection limit of the real-time PCR for the NS1 gene of PPV was 1.00 × 10^2 ^copies/μL. C. Sensitivity of normal PCR. N: Negative Control. M: DNA marker DL2000. Lane 1-9: The standard plasmid was 10-fold serially diluted as template. D. Specificity of the real-time PCR assay. PPV:Positive sample; A-F:Negative control, PCV2, PRV, PRRSV, CFSV, JEV, and H_2_O control.

The standard curve was generated with a range of 6.00 × 10^9 ^to 6.00 × 10^2 ^copies/μL (Figure 1A). The assays were linear in a dilution range of template DNA from 6.00 × 10^2 ^to 6.00 × 10^9 ^copies/μL, with R^2 ^values of 0.996 and reaction efficiencies of 100% for NS1. Quantitative data for Cycling A. FAM are shown in Figure 1B. The amplification product was about 123 bp long, and no false amplification was observed.

### Sensitivity of the real-time PCR

The detection limit of the real-time PCR for the NS1 gene of PPV was 1.00 × 10^2 ^copies/μL(Figure 1B). The conventional PCR assay showed a negative result when the solution was diluted to 1.00 × 10^4 ^copies/μL. These results indicate that, based on direct observation, the sensitivity of the real-time PCR is 100 times greater than that of the conventional PCR (Figure 1C).

### Specificity of the real-time PCR

The real-time PCR gave positive results for the standard plasmid of PPV strains (6.00 × 10^8 ^copies/μL) and negative results for the other porcine viruses involved in reproductive disorders (PCV2, PRV, PRRSV, CFSV, and JEV) and for the sterile water control (Figure 1D).

### Reproducibility of the real-time PCR

When the serially diluted standard plasmid (6.00 × 10^8^, 6.00 × 10^6^, 6.00 × 10^4^, and 6.00 × 10^2 ^copies/μL) was subjected to the real-time PCR, the within-block CV value (four replicate assays for each dilution performed at one time) and the among-block CV value (the means of four replicates from each of four times) were relatively small (Table 1).

**Table 1:** Coefficients of variation for the real-time PCR for PPV-NS1 performed for four concentrations of the standard plasmid at one time (one block with four replications per concentration) or at four different times (four blocks).

Within one block or among four blocks	Concentration of standard plasmid (copies/μL)	n	Ct (mean)	**S.D**.	CV (%)
Within	6.0 × 10^8^	4	17.67	0.38	2.15
Within	6.0 × 10^6^	4	24.38	0.53	2.17
Within	6.0 × 10^4^	4	30.23	0.44	1.46
Within	6.0 × 10^2^	4	37.18	0.36	0.97
Among	6.0 × 10^8^	4	17.35	0.04	0. 21
Among	6.0 × 10^6^	4	24.30	0.14	0. 57
Among	6.0 × 10^4^	4	30.83	0.14	0. 46
Among	6.0 × 10^2^	4	37.80	0.16	0. 42

### Detection of PPV in clinical samples by real-time PCR and conventional PCR

Real-time PCR and conventional PCR were used simultaneously with 41 samples that had been collected from several swine herds for diagnostic purposes. Real-time PCR detected PPV in 32 samples and conventional PCR detected PPV in 11 samples; therefore, they were 100% sensitivity and 100% specificity (Table 2).

**Table 2:** Detection of PRRSV in 41 clinical samples by real-time PCR vs. conventional PCR.

	**Conventional PCR**	
	
	**Positive**	**Negative**
Real-time PCR		
Positive	11^a^	21^c^
Negative	0^b^	9^d^

^a, d ^indicate the number of samples that generated similar data for both assays.

^b, d^indicate the number of samples that generated different data for the assays.

## Discussion

This study describes a real-time PCR assay for PPV based on detection of the NS1 gene. The real-time PCR assay was 100 times more sensitive than conventional PCR. The assay was specific in that it provided positive results with different strains of PPV but negative results with other viruses (PCV2, PRV, PRRSV, CFSV, JEV) associated with reproductive disorders of swine. The real-time PCR assay was also highly reproducible.

The real-time PCR method has several advantages for detection of PPV. First, the real-time PCR is more sensitive than conventional PCR, and high sensitivity is required for early diagnosis of PPV in the clinic. Second, the real-time PCR is faster than conventional PCR because it does not require gel electrophoresis. Third, the TaqMan real-time PCR described here is less likely to produce a false positive than a conventional PCR assay or the SYBR Green I-based real-time PCR. TaqMan-based real-time PCR may be more specific than SYBR Green I-based real-time PCR because the former requires specific probes that bind with PPV DNA template, while the latter uses probes that can bind to any double-stranded DNA, even non-specific amplicons.

Design of the primers and probe was based on the NS1 region of the PPV genome because this region is highly conserved. NS1 protein is believed to be a bifunctional protein with ATPase and helicase activities. In all parvoviral DNAs, both the 5'- and 3'-terminal palindromic sequences act as primers during replication [10].

## Conclusion

In conclusion, the TaqMan real-time PCR assay has been shown to be rapid, sensitive, specific, and reproducible for the detection and quantification of PPV. It should be an excellent tool for laboratory detection of PPV in tissue-culture samples as well as in field samples. The TaqMan real-time PCR assay will be useful for studying the molecular epidemiology of PPV infections in swine populations. The assay will also be very useful for early diagnosis of PPV and therefore for management of PPV.
